# Changes in the Statistical Report

**Published:** 1920-04-10

**Authors:** 


### King Edward's Hospital Fund for London.

### Changes in the Statistical Report.

SPECIAL COMMITTEE APPOINTED.

The General Council of the King's Hospital Fund has adopted the report of the Executive Committee, which states that the Fund's Statistical Report has been enlarged and improved on several occasions since it was introduced in 1904, and it is realised that changes and progress in hospital conditions now call for additions and modifications in the statistics prepared. During the war the report was, in consequence of the need for economy in staff and material, reduced in size by the omission of various tables, and it was thought desirable to make only such other alterations as were absolutely necessary. A considerable number of suggestions have accumulated and have been receiving the attention of the honorary secretaries during recent years, and it is felt that the time has now arrived when the question of again enlarging the report and of making any further necessary and useful changes should be actively taken up

His Royal Highness the Prince of Wales, President, having appointed a special committee to be called the Statistical Report Committee—consisting of the Hon. Sir Arthur Stanley (chairman), Sir Cooper Perry, and Mr. Leonard L. Cohen—to inquire and report on the subject, the Executive Committee recommend that powers be delegated to the Special Committee as follows, viz.:

That until the General Council shall otherwise direct, the Statistical Report Committee shall have the powers of the General Council with respect to the following matters, that is to say: (1) To consider any suggestions for the enlargement or improvement of the Fund's Statistical Report, including those noted by the Fund from time to time and set out in the memorandum dated December 18, 1919. (2) To consider generally what, if any, additions to or improvements in the Fund's Statistical Report would be advisable and practicable. (3) To make such recommendations as may seem to them expedient.

